# Surface Oxygen Injection in Tin Disulfide Nanosheets for Efficient CO_2_ Electroreduction to Formate and Syngas

**DOI:** 10.1007/s40820-021-00703-6

**Published:** 2021-09-06

**Authors:** Tao Chen, Tong Liu, Tao Ding, Beibei Pang, Lan Wang, Xiaokang Liu, Xinyi Shen, Sicong Wang, Dan Wu, Dong Liu, Linlin Cao, Qiquan Luo, Wei Zhang, Wenkun Zhu, Tao Yao

**Affiliations:** 1 National Synchrotron Radiation Laboratory, University of Science and Technology of China, Hefei, 230029 People’s Republic of China; 2 State Key Laboratory of Environmentally Friendly Energy Materials, School of National Defense Science and Technology, Southwest University of Science and Technology, Mianyang, 621010 People’s Republic of China; 3 Institutes of Physical Science and Information Technology, Anhui University, Hefei, 230601 People’s Republic of China; 4 School of Materials, Sun Yat-Sen University, Guangzhou, 510275 People’s Republic of China

**Keywords:** Oxygen injection, Tin disulfide, CO_2_ electroreduction, Formate, Syngas

## Abstract

Surface chemistry modification represents a promising strategy to tailor the adsorption and activation of reaction intermediates for enhancing activity. Herein, we designed a surface oxygen-injection strategy to tune the electronic structure of SnS_2_ nanosheets, which showed effectively enhanced electrocatalytic activity and selectivity of CO_2_ reduction to formate and syngas (CO and H_2_). The oxygen-injection SnS_2_ nanosheets exhibit a remarkable Faradaic efficiency of 91.6% for carbonaceous products with a current density of 24.1 mA cm^−2^ at −0.9 V vs RHE, including 83.2% for formate production and 16.5% for syngas with the CO/H_2_ ratio of 1:1. By *operando* X-ray absorption spectroscopy, we unravel the in situ surface oxygen doping into the matrix during reaction, thereby optimizing the Sn local electronic states. *Operando* synchrotron radiation infrared spectroscopy along with theoretical calculations further reveals that the surface oxygen doping facilitated the CO_2_ activation and enhanced the affinity for HCOO* species. This result demonstrates the potential strategy of surface oxygen injection for the rational design of advanced catalysts for CO_2_ electroreduction.

**Supplementary Information:**

The online version contains supplementary material available at 10.1007/s40820-021-00703-6.

## Highlights

- A surface oxygen-injection strategy is proposed to synergistically modulate the electronic structure of the SnS_2_ nanosheets, thereby regulating the oxophilicity of the catalyst surface.
- The surface oxygen doping facilitates the CO_2_ activation and enhances the affinity for HCOO* species.
- The oxygen-injection SnS_2_ nanosheets exhibit a remarkable Faradaic efficiency of 91.6% for carbonaceous products with a current density of 24.1 mA cm^−2^ at -0.9 V vs RHE.

**Supplementary Information:**

The online version contains supplementary material available at 10.1007/s40820-021-00703-6.

## Graphical abstract

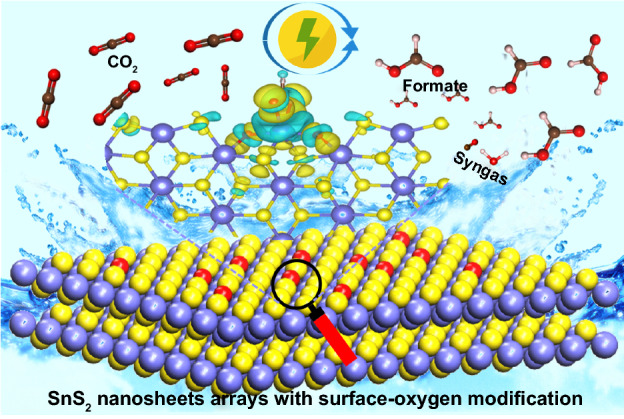

## Introduction

Electroreduction of carbon dioxide (CO_2_RR) into high-value fuels and feedstocks offers a compelling pathway not only to meet the increasing energy demand, but also to alleviate the environmental crisis caused by CO_2_ emissions [1–3]. According to the gross-margin model, formate is considered to be one of the most economically feasible products in the CO_2_RR, which can be widely used as an important raw material in the chemical and pharmaceutical industries, as well as a potential hydrogen carrier and the liquid fuel for proton-exchange membrane fuel cell [4–6]. Up to now, various metal-based electrocatalysts, such as Pd, In, Hg, Pb, Cd, and Sn, have been exploited to achieve the CO_2_ electroreduction to formate [7–13]. Among these electrocatalysts, Sn-based materials have attracted considerable attention due to their advantages of earth abundance, non-toxicity, and low cost. Unfortunately, the catalytic performance of most Sn-based materials is still limited by the high energy barrier for CO_2_ activation, which is usually attributed to the poor stabilization of CO_2_*^−^ intermediates [14–18]. To this end, it is of great significance to develop an efficient and durable Sn-based catalysts for the CO_2_ electroreduction to formate.

Given that the CO_2_ molecule activation is closely related to the number and inherent activity of active sites, many effective strategies have been employed to tailor the active sites of electrocatalysts for enhancing the efficiency of the CO_2_ electroreduction to formate [19–21]. The surface chemistry modification, as a powerful strategy, has attracted great interest in adjusting electronic properties of active sites to target intermediate adsorption energy as well as harvest high selectivity [22–25]. For example, Xie et al. developed a general amino acid modification approach on Cu electrodes for the selective electroreduction of CO_2_ toward hydrocarbons [26]. Previous theoretical calculations have confirmed that the *OCHO binding energy is closely associated with the oxophilicity of the catalyst surface, which can be achieved by modifying the surface of the electrocatalyst with oxygen atom [27]. For instance, Gao et al. reported a phenomenon that partially oxidized atomic cobalt layers effectively adjusted the electronic structure, promoted the activation of CO_2_, and stabilized the relevant key intermediates, thereby enhancing the efficiency of the CO_2_ electroreduction to liquid fuel [28]. As another example, Won et al. prepared hierarchical Sn dendrites and found that the natural oxygen content is closely related to the stability of CO_2_*^−^ intermediates and the selectivity of formate [29]. To improve the catalytic performance of Sn-based materials, oxygen modification is a promising strategy to regulate the surface oxophilicity of the catalysts and further manipulate their electronic structure. In fact, most of the catalysts with surface chemical modification have undergone structural evolution of the active phase under operation conditions, leading to deviations in the understanding the nature of the active site. Therefore, monitoring the structural evolution of Sn-based catalysts with surface oxygen modification under realistic working conditions is crucial for understanding the nature of the active phase and the rational design of targeted CO_2_RR catalysts.

Herein, the SnS_2_ nanosheets arrays on the carbon paper with surface oxygen modification were rationally designed under the guidance of density function theory (DFT) to effectively electroreduce CO_2_ into formate and syngas (CO and H_2_). The introduction of oxygen into the surface of SnS_2_ nanosheets achieved the exposure of Sn active sites and optimal Sn electronic states, thereby enhancing the adsorption and activation of CO_2_. Specifically, the SnS_2_ nanosheets with surface oxygen modification exhibit a remarkable Faradaic efficiency of 91.6% for carbonaceous products at −0.9 V vs RHE, including 83.2% for formate production and 16.5% for syngas with the CO/H_2_ ratio of 1:1. Operando X-ray absorption spectroscopy unravels that the in situ surface oxygen doping into the matrix under working conditions effectively changes the local electronic state of Sn, thereby providing an optimized electronic structure to improve CO_2_RR performance. In addition, operando synchrotron radiation infrared spectroscopy and DFT calculations further confirm that the local electronic state of Sn is manipulated through surface oxygen modification, thereby promoting the CO_2_ activation and enhancing the affinity for HCOO* species.

## Experimental Section

The experimental details are provided in Supporting Information (SI). This section briefly summarizes the synthesis measurements.

In a typical synthesis of SnS_2-x_O_x_/CC, 5 mmol of SnCl_4_·5H_2_O and 15 mmol of thioacetamide were dissolved in 40 mL of deionized water. The mixture and carbon paper (2 × 2) were then transferred into a Teflon-lined stainless-steel autoclave, followed by being heated to 190 °C for 8 h. After the mixture was cooled down naturally to room temperature, the SnS_2_/CC was washed by water three times and ethanol twice to remove any possible ions, followed by being dried under vacuum at 60 °C for 12 h. The SnS_2-x_O_x_/CC was prepared by placing the SnS_2_/CC in the muffle furnace that had been heated at 300 °C for several minutes.

## Results and Discussion

### Preparation and Characterization of SnS_2-x_O_x_/CC

**Fig. 1:**
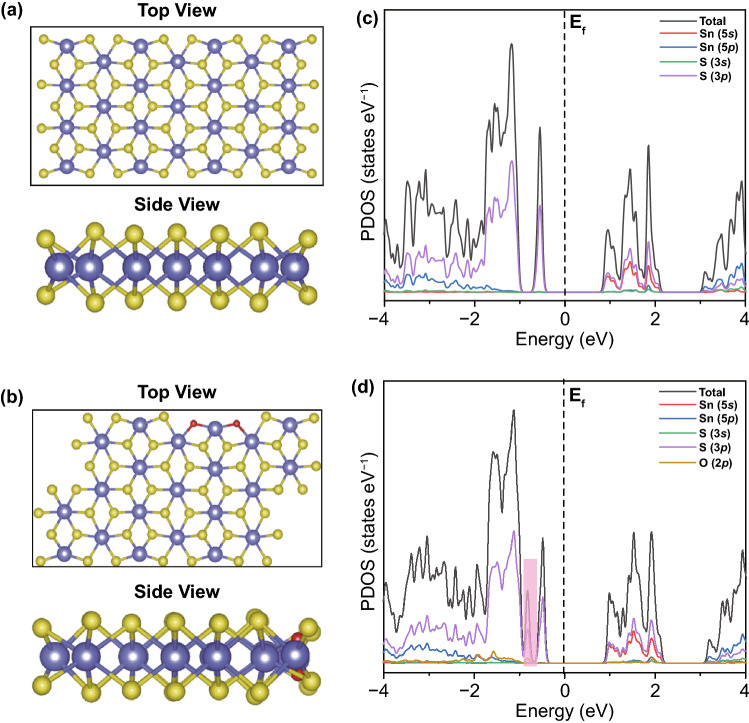
Top view and side view of the model for **a** pristine SnS_2_ and **b** SnS_2-x_O_x_. Calculated DOS of **c** SnS_2_ and **d** SnS_2-x_O_x_ slabs

At first, to gain insight into the effect of surface oxygen-injection engineering on electronic properties of SnS_2_ nanosheets, we conducted DFT calculations by using the SnS_2_ slab with/without oxygen injection as the models (Fig. 1a, b). Compared with the pristine SnS_2_, the surface oxygen injection leads to a new additional state near the Fermi level (Fig. 1c, d), which is beneficial to manipulate the local electronic structure of Sn and expose the active site of Sn at the edges. Notably, O 2*p* states also contributed the unoccupied part of these levels, making them serve as the highly catalytically active sites. Furthermore, the electronic localization functions (ELF) exhibit that the charge density is mainly derived from the S atoms for both the SnS_2_ with/without oxygen injection (Fig. S1). Owing to the introduction of oxygen atoms, the electron density of the whole system has undergone distinctly change, further indicating surface oxygen injection effectively tailors the local electronic structure of Sn.

**Fig. 2:**
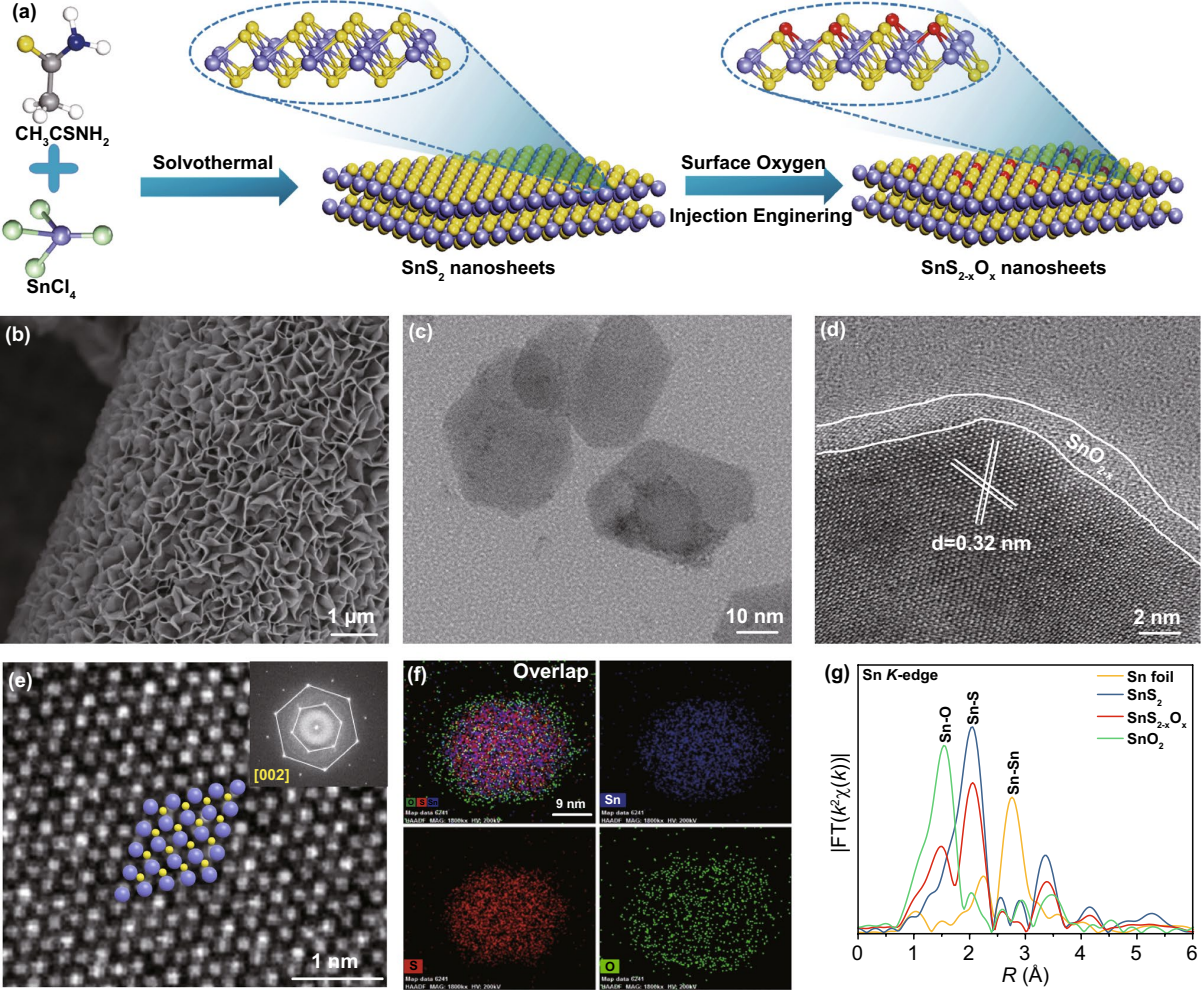
**a** Schematic illustration of the manufacture of SnS_2-x_O_x_ nanosheets. **b** SEM image of SnS_2-x_O_x_/CC. **c** TEM image of SnS_2-x_O_x_ nanosheets. **d, e** HRTEM image and the homologous FFT pattern of SnS_2-x_O_x_ nanosheets (inset in **e**). **f** HAADF-STEM and STEM-EDX elemental mapping images of an individual SnS_2-x_O_x_ nanosheet. **g** Fourier transform spectra of Sn *K*-edge for Sn foil, SnS_2_, SnS_2-x_O_x_, and SnO_2_ from EXAFS

Then, the SnS_2_ nanosheets arrays with partially oxidized surface on the carbon paper (denoted as SnS_2-x_O_x_/CC) were prepared, as schematically illustrated in Fig. 2a. Specifically, the pristine SnS_2_ nanosheets arrays were directly grown on the carbon paper by a simple hydrothermal method. Afterward, the SnS_2-x_O_x_/CC was further synthesized by the low-temperature calcination of the as-prepared SnS_2_/CC under the air atmosphere. The morphology of the SnS_2-x_O_x_/CC was characterized by scanning electron microscopy (SEM) and transmission electron microscopy (TEM). As shown in Fig. 2b, the final products present a hierarchical nanosheets arrays composed of SnS_2-x_O_x_ nanosheets and flexible carbon paper. The TEM images of the SnS_2_ and SnS_2-x_O_x_ took the nanosheet morphology (Figs. 2c and S2a), whereas the SnS_2_ nanosheets were completely oxidized into SnO_2_ nanoplatelets (Fig. S3). The high-resolution transmission electron microscopy (HRTEM) image in Fig. 2d shows that the SnS_2-x_O_x_ lattice fringes with an interplanar distance of 0.32 nm indexed to the (002) facets of SnS_2_, confirming the as-obtained SnS_2-x_O_x_ nanosheets retain its pristine crystal structure (Fig. S2b, c) [30]. Besides, there is an obvious circle of amorphous layer at the edge of the SnS_2-x_O_x_ nanosheet, which is attributed to the partial oxidation on the surface of the SnS_2_ nanosheet. In addition, the homologous fast Fourier transform (FFT) pattern indicates the SnS_2_ phase recorded from [002] orientation (inset in Fig. 2e). The element of O was uniformly distributed on the whole SnS_2-x_O_x_ nanosheet which can be further confirmed by the high-angle annular dark-field energy-dispersive X-ray spectroscopy (HAADF-EDS) elemental mapping and EDS spectrum (Figs. 2f and S4).

To further investigate the phase composition and electronic structure of the SnS_2-x_O_x_/CC, we performed X-ray diffraction (XRD) and X-ray photoelectron spectroscopy (XPS) measurement. As evidenced by the XRD patterns in Fig. S5, the SnS_2_/CC and SnS_2-x_O_x_/CC exhibited the diffraction peaks at 30.74°, 32.09°, and 44.98°, which were indexed to the (200), (101), and (211) planes of hexagonal SnS_2_ (JCPDS No. 23–0677) [31]. Remarkably, no additional peaks corresponding to the phases of SnO_2_ could be found, indicating that the surface oxygen injection did not change the crystalline phase of SnS_2_. We further carried out the XPS measurements to clarify the form of O existing in SnS_2-x_O_x_/CC. As disclosed by XPS survey spectra, a weak signal of O was recorded in SnS_2-x_O_x_/CC, further confirming successful introduction of O (Figs. S6 and S7). In addition, the peaks at 495.3 and 486.8 eV were attributed to Sn 3*d*_3/2_ and Sn 3*d*_5/2_ of SnS_2-x_O_x_/CC, respectively (Fig. S8) [32]. Compared with the SnS_2_/CC, the Sn 3d_3/2_ and 3d_5/2_ peaks for SnS_2-x_O_x_/CC shifted to higher binding energies, due to the larger electronegativity of O than that of S.

The surface-sensitive synchrotron radiation soft X-ray absorption structure (XAS) was further employed to investigate the changes in the local electronic structure of SnS_2_ caused by surface oxygen-injection engineering. As shown in Fig. S9, O *K*-edge XAS spectra for SnS_2-x_O_x_/CC and pure SnO_2_/CC displayed similar shapes, implying SnO_2_ species were formed on the surface of SnS_2-x_O_x_/CC, further confirming the surface oxygen injection successfully replaced the S atoms. In addition, S *L*-edge XAS spectra exhibited the two characteristic peaks located at 163.3 eV (S-Sn π* peak) and 166.6 eV (S-Sn σ* peak) observed in pure SnS_2_/CC and the SnS_2-x_O_x_/CC (Fig. S10). Compared with pristine SnS_2_/CC, the relative strength of S-Sn for SnS_2-x_O_x_/CC was slightly reduced, which was attributed to the substitution of partial S atoms on the surface of SnS_2-x_O_x_/CC by O atoms. Meanwhile, the X-ray absorption near-edge structure (XANES) measurement was employed to further investigate the effect of surface oxygen injection. Compared with pristine SnS_2_/CC, the white line peak of the SnS_2-x_O_x_/CC shifted to the high-*E* region, due to the electronegativity of O being greater than that of S, in consistent with the results of Sn 3*d* XPS spectrum (Fig. S11). Given that the white line peak of Sn *K*-edge intensity originating from the transition of the 1* s* to 5*p* orbital, the increase in the white line peak intensity after surface oxygen injection indicates the increases in the possibility of electron transition from the 1* s*-5*p* orbital. The above results reveal the surface oxygen injection effectively manipulates the local electronic structure of Sn.

Furthermore, the Fourier transform (FT) *k*^2^-weighted extended XAFS (EXAFS) spectrum of the Sn *K*-edge was employed to further reveal the effect of surface oxygen injection on the local electronic structure of Sn at the atomic level. Considering the surface oxygen injection into the SnS_2_ nanosheets, we performed out least-squares EXAFS curve fitting analysis for Sn by considering two backscattering paths, including Sn–S and Sn–O. Compared with the SnS_2_/CC, the Sn *K*-edge FT-EXAFS curve for SnS_2-x_O_x_/CC presented a new peak at 1.49 Å, which is ascribed to the Sn–O coordination (Fig. 2a) [33]. By quantitative EXAFS curve fitting analysis, the coordination number of Sn-S for SnS_2-x_O_x_/CC is confirmed to be 4.3, smaller than that of pristine SnS_2_/CC (6.0), and the coordination number of Sn–O is verified to be 2.1, further confirming surface oxygen injection successfully replaced the S atoms (Table S1 and Fig. S12). Moreover, the wavelet transform (WT) of Sn *K*-edge EXAFS oscillations exhibited the intensity maxima at 4.3 Å^−1^ and around 8.2 Å^−1^ of SnS_2-x_O_x_/CC, which associate with Sn–O and Sn–S contributions, respectively (Fig. S13). Taken together, the successful injection of surface oxygen effectively manipulated the local electronic structure of SnS_2_.

### Electrocatalytic CO_2_RR Performances of the SnS_2-x_O_x_/CC

Surface oxygen-injection engineering provides a potential prospect for enhancing the CO_2_ electroreduction. The electrocatalytic CO_2_ reduction activities of the three Sn-based catalysts were evaluated using a three-electrode H-cell in CO_2_-saturated 0.5 M KHCO_3_. The linear sweep voltammetric (LSV) curves in Fig. S14 revealed that the SnS_2-x_O_x_ nanosheets exhibited higher current density than that of pristine SnS_2_ nanosheets, confirming the injection of oxygen effectively enhanced the electrocatalytic activity of SnS_2_/CC. Particularly, the geometrical current density of SnS_2-x_O_x_/CC achieved 19.68 mA cm^−2^, which was 2.7 times higher than that of pristine SnS_2_/CC at overpotential of −0.8 V vs RHE (Fig. 2a). For these three Sn-based catalysts, H_2_, CO, and formate were the main catalytic products, which are quantified by online gas chromatography and ^1^H NMR analysis (Fig. S15). Figure 2b exhibits partial current density for carbonaceous products (CO and formate), respectively. At all applied potentials, the SnS_2-x_O_x_/CC presented the largest current density among the three electrocatalysts, demonstrating the high activity for CO_2_ electroreduction. As shown in Fig. 2c, the SnS_2-x_O_x_/CC displayed the highest Faradaic efficiency (FE) for carbonaceous products among the three electrocatalysts, while the pristine SnS_2_/CC exhibited the lowest FE value. At −0.9 V vs RHE, the SnS_2-x_O_x_/CC exhibited the FE of 91.8% for carbonaceous products, including the FE of 83.5% for formate production and the FE of 16.5% for syngas with the H_2_/CO ratio of 1:1. It is worth noting that such syngas ratio is optimal for multiple chemical synthesis (e.g., Fischer–Tropsch synthesis, fermentation and alcohol synthesis, and hydroformylation processes). Furthermore, the as-prepared SnS_2-x_O_x_/CC displayed an excellent durability for 10-h potentiostatic test with the less than 3% decay in current density, together with the FE for formate and CO keeping steady at −0.9 V vs RHE (Fig. 2d). The above results demonstrate that the SnS_2-x_O_x_/CC represents a promising catalyst for persistently producing formate and syngas toward CO_2_RR.

**Fig. 3:**
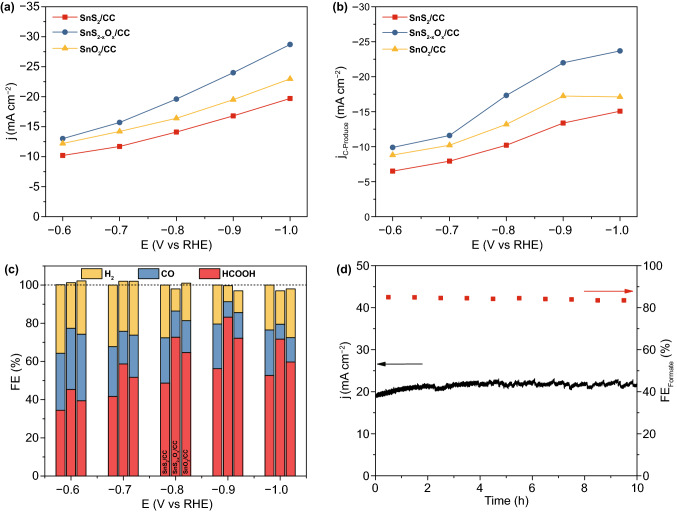
**a** Geometrical current densities over SnS_2_/CC, SnS_2-x_O_x_/CC, and SnO_2_/CC. **b** Current densities for carbonaceous product (C-product) over SnS_2_/CC, SnS_2-x_O_x_/CC, and SnO_2_/CC. **c** Faradaic efficiencies for formate, H_2_, and CO production over SnS_2_/CC, SnS_2-x_O_x_/CC, and SnO_2_/CC. **d** Plot of geometrical current density (*j*) and Faradaic efficiencies for C-product versus time over the SnS_2-x_O_x_/CC at a constant potential of −0.9 V vs RHE

Inspired by surface oxygen injection to improve the CO_2_ electroreduction performance of SnS_2_/CC, we studied the microscopic reaction kinetics of the pristine SnS_2_/CC and SnS_2-x_O_x_/CC. Based on cyclic voltammogram measurements at different scan rates, the double-layer capacitance (*C*_dl_) value increased from 3.27 mF cm^−2^ of the pristine SnS_2_/CC to 3.75 mF cm^−2^ of the SnS_2-x_O_x_/CC, indicating that surface oxygen injection effectively increases the electrochemical active surface area (ECSA) of the electrocatalysts (Figs. S16 and S17). Given that the ECSA of the electrocatalysts is positively correlated with the active sites, we have reason to believe that the surface oxygen modification effectively exposes the active site of Sn. The Tafel plots were further employed to verify the rate-limiting step of the Sn-based catalysts in the CO_2_RR process. The Tafel slopes of the Sn-based catalysts were all close to 118 mV dec^−1^, demonstrating that the activation of CO_2_ served as the rate-limiting step (Fig. S18) [34,35]. In addition, the Nyquist plots were used to confirm the facilitated electron transfer process [36]. The SnS_2-x_O_x_/CC displayed the charge transfer resistance (R_CT_) of 12.1 Ω, which was smaller than that (15.8 Ω) of SnS_2_/CC (Fig. S19). Therefore, surface oxygen injection effectively accelerates the charge transfer process of SnS_2_/CC during the CO_2_RR (Fig. 3).

### *Operando* X-ray Absorption Spectroscopy Study

**Fig. 4:**
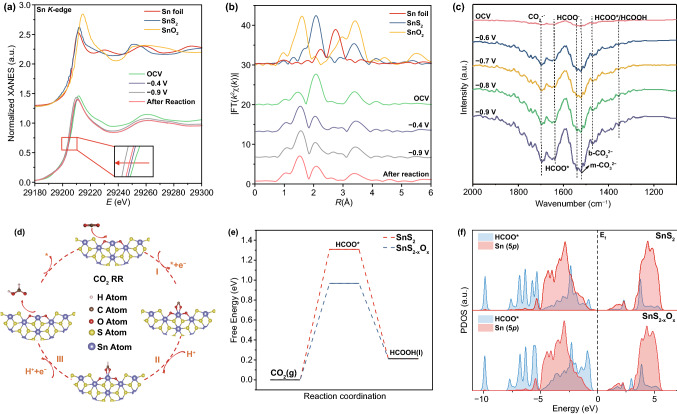
**a** *Operando* XANES spectra at the Sn *K*-edge. **b** *Operando k*^2^-weighted FT spectra at the Sn *K*-edge. **c** *Operando* SR-FTIR spectra of the SnS_2-x_O_x_/CC under the working condition. **d** Schematic of the whole CO_2_RR mechanism on SnS_2-x_O_x_ nanosheets. **e** Gibbs free energy diagrams for CO_2_ reduction to HCOOH on SnS_2_ and SnS_2-x_O_x_ slabs. **f** Projected density of states (PDOS) of HCOO* adsorbed on the SnS_2_ and SnS_2-x_O_x_ slabs. The * represents an adsorption site

Given that the bulk phase stability of transition metal chalcogenides with heat treatment is destroyed, the bulk phase is in a relatively unstable state [37]. Based on the equilibrium theory of crystalline chemistry, the catalyst in the electrolyte driven by both energetical and kinetical force will tend to freely optimize the structure of the entire bulk, so that the bulk tends to a relatively stable state [38–41]. Therefore, we employed *operando* XAFS measurements to monitor the structural evolutions of the SnS_2-x_O_x_/CC under realistic working conditions. Figure 4a shows the *operando* Sn *K*-edge XANES spectra at different applied potentials, along with the data for Sn foil, SnS_2_, and SnO_2_ as references. When cathodic potentials were applied, the absorption edge of Sn *K*-edge XANES spectra shifted toward low-*E* side compared to the case of the open-circuit condition, indicating the decrease in the Sn valence state during CO_2_RR process. Furthermore, when a cathodic potential of −0.9 V versus RHE was applied, the white line peak intensity was significantly increased in relation to the case (−0.4 V versus RHE), indicating more 5*p* electrons participate in the reaction. After the reaction, the white line peak approximately returned to the state (−0.4 V versus RHE), further confirming that the SnS_2-x_O_x_/CC catalyst undergone in situ reconstruction during the reaction and tended to form a relatively stable state.

Furthermore, the EXAFS was further employed to reveal the atomic reconstruction of the SnS_2-x_O_x_/CC catalyst under working conditions (Figs. 4b and S20). At first sight, the Fourier transform curves of SnS_2-x_O_x_/CC displayed a significantly dampening in the Sn-S coordination peak and a heightening in the Sn-nonmetallic coordination peak under working condition. Specifically, at the applied potential of −0.4 V versus RHE before the occurrence of CO_2_RR, the EXAFS fitting results showed that the Sn–O coordination number increased from 2.5 to 3.6, which may be ascribed to the further doping of surface oxygen into the SnS_2_ lattice during the reaction. To further verify the above conjecture, we performed XRD on the SnS_2-x_O_x_/CC after reaction. As expected, the intensity of the diffraction peaks of SnS_2_ was significantly reduced after the reaction, and the characteristic peak of SnO_2_ appeared in the SnS_2-x_O_x_/CC after reaction (Fig. S21). Moreover, at the potential of -0.9 V versus RHE during CO_2_RR, Sn–O coordination number arose from 3.6 to 4.2 and the Sn-S coordination number remained unchanged, which may be attributed to the adsorption of the reaction intermediate species. When the cathode potential was removed, the Sn–O coordination number recovered to the state at −0.4 V, while the coordination number of Sn–S remained unchanged (Table S2). The above results indicated that the SnS_2-x_O_x_/CC had undergone dynamic surface reconstruction and surface oxygen doping plays a critical role under reaction conditions.

### *Operando* Synchrotron Radiation Fourier Transform Infrared Spectroscopy Study

*Operando* synchrotron radiation Fourier transform infrared spectroscopy (SR-FT-IR) was further employed to investigate the catalytic mechanism for the well-designed SnS_2-x_O_x_/CC during the CO_2_RR. All SR-FTIR spectra were recorded with the electrocatalysts at CO_2_RR catalytic process (open circuit, − 0.6, − 0.7, − 0.8, and − 0.9 V) to reveal the production and transformation of key intermediates. As displayed in Fig. 4c, the monodentate carbonate groups (m-CO_3_^2−^) appeared at the peaks of ~ 1520 cm^−1^, demonstrating that more CO_2_ was adsorbed on the surface of electrocatalyst with the decrease in applied voltage. Meanwhile, a new characteristic peak appeared in the SR-FTIR spectra of ~ 1694 cm^−1^ (CO_2_·^−^ radicals) and the intensity of peak continually increased as the applied potentials decreased, indicating that the CO_2_ molecules adsorbed on the catalyst surface were activated to CO_2_·^−^ radicals during the reaction ^[[42]]^. Meanwhile, as the cathode potential decreases, the peak intensity at ~ 1541 cm^−1^ (HCOO^−^) increased further confirming the excellent proton trapping ability of CO_2_·^−^ radicals [33]. The peaks at ~1354 cm^−1^ and ~1660 cm^−1^ is ascribed to the symmetry vibration of the HCOO* intermediates, which corresponds to the key intermediates or the products for CO_2_ electroreduction [43]. Based on the above-mentioned operando SR-FTIR analysis, the pathway of electroreduction from CO_2_-to-HCOOH conversion by the SnS_2-x_O_x_/CC could be proposed as the following reactions (Fig. 4d):

1 $${\text{Step 1}}:{\text{ CO}}_{2} \left( {\text{g}} \right){\mkern 1mu} + {\text{ e}}^{ - } + ^{*} \to {\text{CO}}_{2}^{*} - \left( {{\text{Activation process}}} \right)$$

2 $${\text{Step 2}}:{\text{ CO}}_{2} ^{* - } + {\text{ HCO}}_{3}^{ - } \left( {{\text{aq}}} \right) \to {\text{HCOO}}^{*} + {\text{ CO}}_{3}^{{2 - }} \left( {{\text{Surface reaction}}} \right)$$

3 $${\text{Step 3}}:{\text{ HCOO}}^{*}{\mkern 1mu} + {\text{ HCO}}_{3}^{ - } \left( {{\text{aq}}} \right)^{{}} + {\text{ e}}_{{}}^{ - } \to {\text{HCOOH }}\left( {\text{l}} \right){\mkern 1mu} + {\text{ CO}}_{3}^{{2 - }} ({\text{Desorption process}})$$

### Density Functional Theory (DFT) Calculations

DFT calculations were employed to elucidate the catalytic contribution from partial oxidation at SnS_2-x_O_x_ for CO_2_RR. The models for pristine SnS_2_ and SnS_2-x_O_x_ were chosen for the simulation. Figure S22 shows optimized adsorption configurations of HCOO* intermediates on the armchair edges of the pristine SnS_2_ slab and SnS_2_O_x_ slab with distinguishable Sn-O distances. from the material [44]. Specifically, the Sn–O bond length (d_Sn-O_) is 2.28 Å for SnS_2_, while the bond length d_Sn-O_ for SnS_2-x_O_x_ is reduced to 2.24 Å, implying that the surface oxygen injection effectively enhances the binding to the HCOO* intermediate. Besides, DFT calculations were further conducted on the Gibbs free energy (ΔG) with multiple elementary reaction steps over SnS_2_ with/without oxygen injection. As exhibited in Fig. 4e, for both SnS_2_ and SnS_2-x_O_x_, the formation of HCOO* is further confirmed to be the rate-limiting step for formate, which is consistent with the results of Tafel slopes. For the SnS_2-x_O_x_ slab, the ΔG for HCOO* formation (ΔG_HCOO*_) was calculated to be 0.97 eV, which is much lower than that for pristine SnS_2_ slab (1.31 eV), indicating surface oxygen injection enhanced the activation of CO_2_ and correspondingly facilitated the formation of HCOO*. To gain an in-depth insight into the nature of surface oxygen doping enhancing the intrinsic activity of SnS_2_, we calculated the projected density of state of HCOO* absorbed SnS_2_ and SnS_2-x_O_x_ (Fig. 4f). In the HCOO* PDOS, the dominant features are that HCOO* exhibits strong interaction with the valence band region of SnS_2_ and SnS_2-x_O_x_, which leads to strong chemical adsorption. Notably, the state density of HCOO* overlaps more with the orbital of Sn (5*p*) in SnS_2-x_O_x_ with regard to that in SnS_2_, and the higher occupied state of HCOO* is near the Fermi level, indicating that HCOO* has a stronger interaction with SnS_2-x_O_x_, which is consistent with the calculation result of ΔG _HCOO*_. Furthermore, the charge density differences calculations also show that more electrons gather around the adsorption site in SnS_2-x_O_x_, indicating that the surface oxygen injection makes the SnS_2_ edges exhibit a stronger affinity for HCOO* species (Fig. S23). The above results confirm that surface oxygen injection alters the local electronic structure of Sn atom with optimal ΔG_HCOO*_ to effectively facilitate the production of formate over CO_2_RR. Particularly, the above theoretical calculation results are consistent with the previous experimental results.

## Conclusions

In conclusion, we developed SnS_2_ nanosheets with surface oxygen modification for CO_2_ electroreduction to formate and syngas (CO and H_2_). The surface oxygen-injection engineering achieved exposure of Sn active site and optimal Sn electronic states, thereby enhancing the adsorption and activation of CO_2_. Surface oxygen injection on SnS_2_ nanosheets significantly improved electrocatalytic activity and selectivity of CO_2_ reduction to formate and syngas (CO and H_2_). Specifically, at −0.9 V vs RHE, the SnS_2-x_O_x_/CC exhibits the highest FE of 91.6% for carbonaceous products, including the FE of 83.2% for formate production and the FE of 16.5% for syngas with the H_2_/CO ratio of 1:1. Moreover, the as-prepared SnS_2-x_O_x_/CC displays an excellent durability for 10-h potentiostatic test with less than 4% decay in current density. *Operando* XAS unravels that the in situ surface oxygen doping into the matrix under working conditions effectively modulates the Sn local electronic state. *Operando* SR-FTIR and DFT calculations reveal that the surface oxygen doping enhanced the affinity for HCOO* species by manipulating the Sn electronic states and accelerated the CO_2_ activation. This work opens a span-new door for the design of advanced catalysts for CO_2_ electroreduction.

## Supplementary Information

Below is the link to the electronic supplementary material.

Supplementary file1 (PDF 1526 kb)
