# Supplementary material for: Surface Oxygen Injection in Tin Disulfide Nanosheets for Efficient CO2 Electroreduction to Formate and Syngas
Source: Nanomicro Lett. 2021 Sep 6;13:189. doi: 10.1007/s40820-021-00703-6 (PMC8421506; doi:10.1007/s40820-021-00703-6)
Supplement: Supplementary file 1 — Supplementary file1 (PDF 1526 kb) [file 40820_2021_703_MOESM1_ESM.pdf]

Supporting Information for

Surface Oxygen-Injection in Tin Disulfide Nanosheets for Efficient CO_2 Electroreduction to Formate and Syngas

Tao Chen[1, 2, #], Tong Liu[1, #], Tao Ding[1], Beibei Pang[1], Lan Wang[1, 2], Xiaokang Liu[1], Xinyi Shen[1], Sicong Wang[1], Dan Wu[1], Dong Liu[1], Linlin Cao[1], Qiquan Luo[3], Wei Zhang[1, 4, *], Wenkun Zhu[2, *], Tao Yao[1, *]

[1]National Synchrotron Radiation Laboratory, University of Science and Technology of China, Hefei 230029, P. R. China

[2]State Key Laboratory of Environmentally Friendly Energy Materials, School of National Defense Science and Technology, Southwest University of Science and Technology, Mianyang, 621010, P. R. China

[3]Institutes of Physical Science and Information Technology, Anhui University, Hefei, 230601, P. R. China

[4]School of Materials, Sun Yat-sen University, Guangzhou, 510275, P. R. China

[#]Tao Chen and Tong Liu contributed equally to this work.

*Corresponding authors. E-mail: zhuwenkun@swust.edu.cn (Wenkun Zhu); zwei2319@mail.ustc.edu.cn (Wei Zhang); yaot@ustc.edu.cn (Tao Yao)

S1 Chemicals and Materials

The tin chloride pentahydrate ($SnCl_4{\cdot}5H_2O$, ≥99.0%), thioacetamide (C_2H_5NS, ≥99.0%), potassium bicarbonate ($KHCO_3$, ≥99.5%), and ethanol (C_2H_5OH, ≥99.7%) were all purchased from Sinopharm Chemical Reagent Co. Ltd. (Shanghai, China). Graphite powder (XF011 7782-42-5) and Carbon black (XFI15 7440-44-0) were obtained from XFNANO Materials Tech Co., Ltd. All the chemicals were used without further purification. Ultrapure Millipore water (18.2 MΩ) was used in all experiments.

S2 Electrochemical Measurements

Electrochemical measurements were performed in a three-electrode system at an electrochemical station (CHI660E). $SnS_{2-x}O_x$/CC doesn't require any treatment and can be used as a supporting electrode for electrochemical carbon dioxide reduction directly. Controlled potential electrolysis of CO_2 was conducted in an H-cell (separated by Nafion 115) containing 75 mL of 0.5 M $KHCO_3$ electrolyte at room temperature and under atmospheric pressure. The platinum network and Ag/AgCl electrode were used as the counter and reference electrodes, respectively. Gas products were analyzed by a thermal conductivity detector (TCD) (for H_2 and CO) and a flame ionization detector (FID) (for alkanes and alkenes). Quantification of the products was performed using standard calibration gases. Liquid products were analyzed by quantitative 1H NMR spectroscopy with water suspension, and using dimethyl sulphoxide (DMSO) as an internal standard.

S3 Characterizations

The TEM, HRTEM, HAADF-STEM, and EDX mapping of the SnS_2/CC, $SnS_{2-x}O_x$/CC and SnO_2/CC were carried out on a JEOL ARM-200F field-emission transmission electron microscope operating at 200 kV accelerating voltage. XRD patterns was recorded by using a Philips X'Pert Pro Super diffractometer with Cu-Kα radiation (λ=1.54178 Å). XPS measurement was performed on a VG ESCALAB MK II X-ray photoelectron spectrometer with an exciting source of Mg Kα = 1253.6 eV.

S4 EXAFS Experimental Details

The Sn *K*-edge (29200 eV) XAFS spectra were performed at BL14W1 station in Shanghai Synchrotron Radiation Facility (SSRF), China. The storage rings of SSRF were operated at 3.5 GeV with the maximum current of 210 mA. During XAFS measurements, we calibrated the position of the absorption edge (E_0) using Sn foil. And all the XAFS data were collected during one period of beam time. Each spectrum was measured three times to ensure the repeatability of the data (the positions of E_0 were almost the same during the multiple scans). The position of E_0 is defined as the point corresponding to the maximum value in the derivative curves of the XANES spectra.

S5 DFT Calculations

The calculations were performed within the Density Functional Theory (DFT) framework implanted in Vienna ab initio Simulation Package (VASP)[2]. The interaction between ions and electrons was described in the Projector Augmented Wave (PAW) Method[4]. The electron exchange and correlation energy was described using the generalized gradient approximation-based Perdew–Burke–Erzenhorf (PBE) functional[3]. The models of armchair edges SnS_2 and $SnS_{2-x}O_x$ with a 1 × 3 × 3 supercell were chosen for the calculation. And all the atoms were fully relaxed during the calculations. A sufficiently large vacuum region of 15 Å was used for all the models to ensure the periodic images were well separated. The Brillouin-zone integrations were carried out using Monkhorst-Pack grids of special points. A gamma-centered (1 × 3 × 1) k-point grid was used for SnS_2 and $SnS_{2-x}O_x$ supercell. To obtain the accurate structure, The plane-wave cutoff energy was set up to 500 eV. The force convergence was set to be <0.02 eV $Å^{-1}$, and the total energy convergence was set to be $<10^{-5}$ eV.

The free energy of the adsorbed state was calculated as follows based on the adsorption energy:

$$\Delta G_{HCOO*} = \Delta E_{HCOO*} + \Delta E_{ZPE} + U_{(T)} - T\Delta S$$

where ΔE_{HCOO*} is the adsorption energy of hydrogen directly obtained from DFT calculations, ΔE_{ZPE} is the zero-point energy, $U_{(T)}$ is the heat capacity correction energy, and T is the temperature (T = 298.15 K), ΔS is the change in entropy. Herein, the Gibbs energy is corrected by using the VASPKIT code [6].

S6 Supplementary Figures and Tables

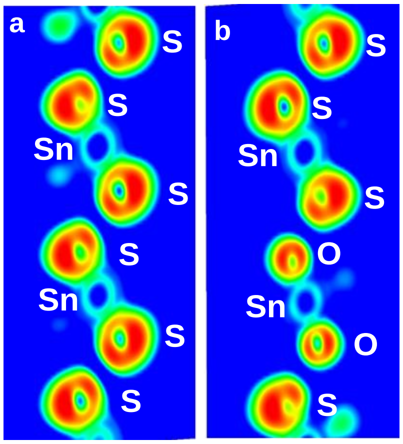

Fig. S1 Electron Localization Function (ELF) for pristine SnS_2 slab (**a**) and $SnS_{2-x}O_x$ slab (**b**)

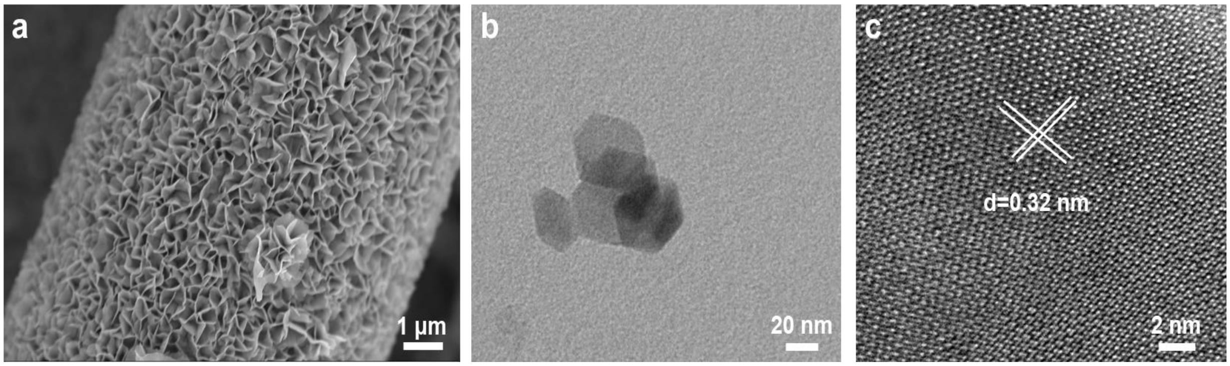

Fig. S2 (**a**) Typical SEM images of SnS_2/CC, (**b**) Typical TEM images of SnS_2 nanosheets, (**c**) HRTEM image of pristine SnS_2 nanosheets

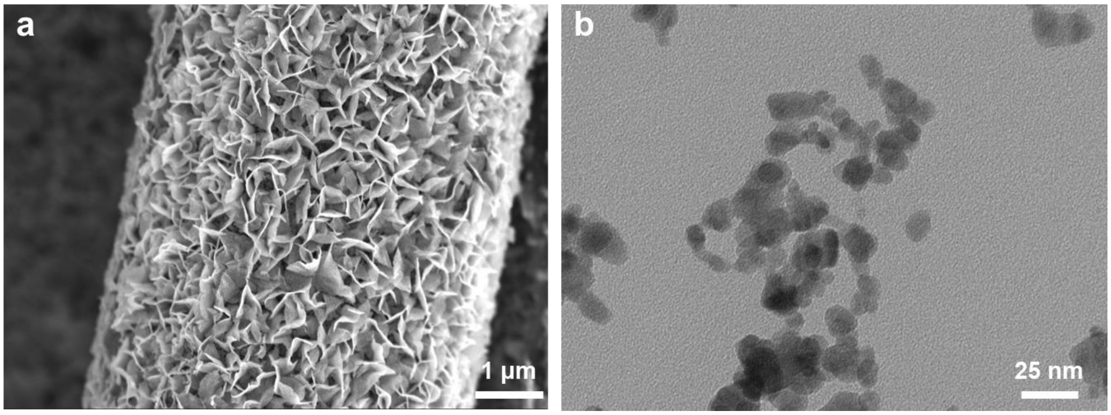

Fig. S3 (**a**) Typical SEM images of SnO_2/CC, (**b**) Typical TEM images of SnO_2 nanoplatelets

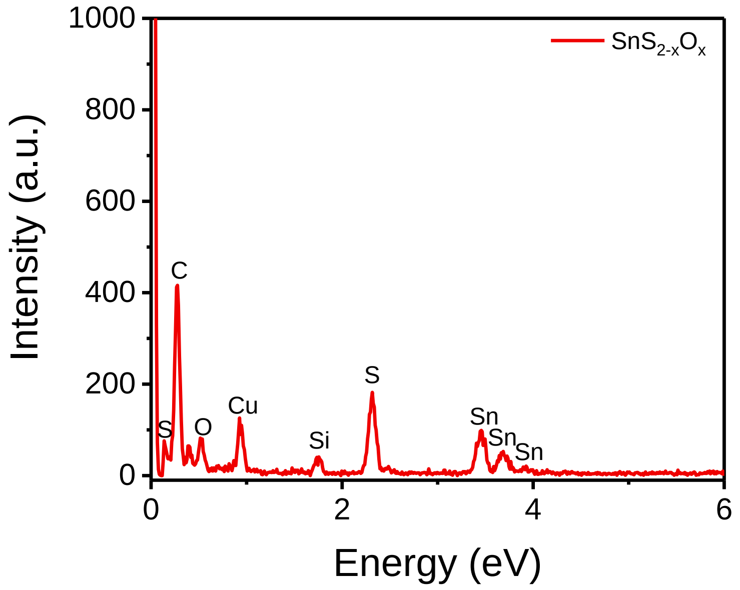

Fig. S4 EDX spectrum of $SnS_{2-x}O_x$ nanosheets

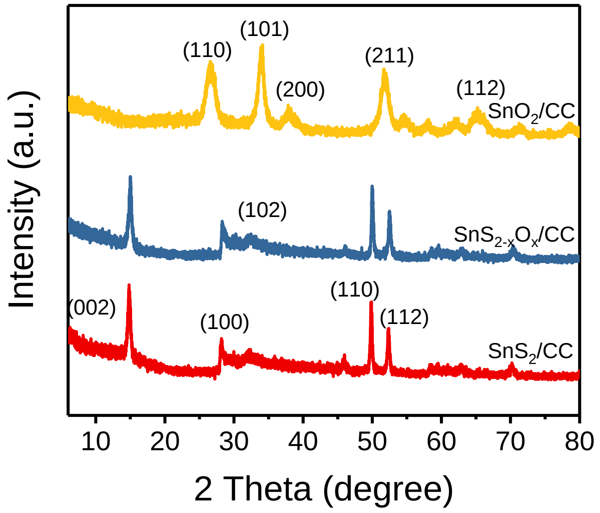

Fig. S5 XRD patterns of pristine SnS_2/CC, $SnS_{2-x}O_x$/CC, and SnO_2/CC

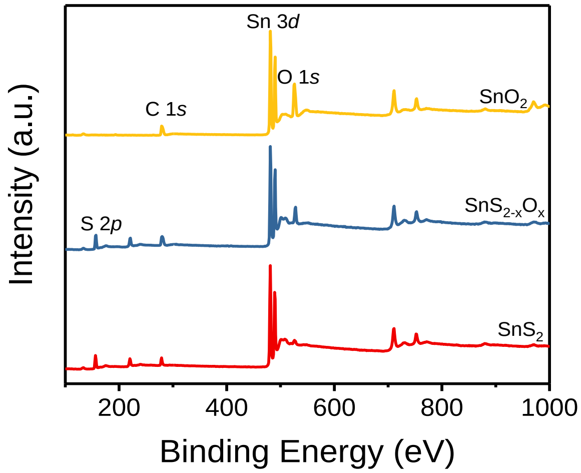

Fig. S6 XPS survey spectra of pristine SnS_2/CC, $SnS_{2-x}O_x$/CC, and SnO_2/CC

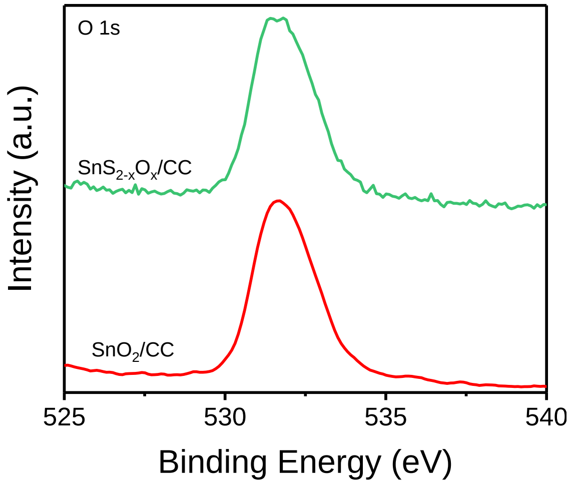

Fig. S7 O 1*s* XPS spectra of $SnS_{2-x}O_x$/CC and SnO_2/CC

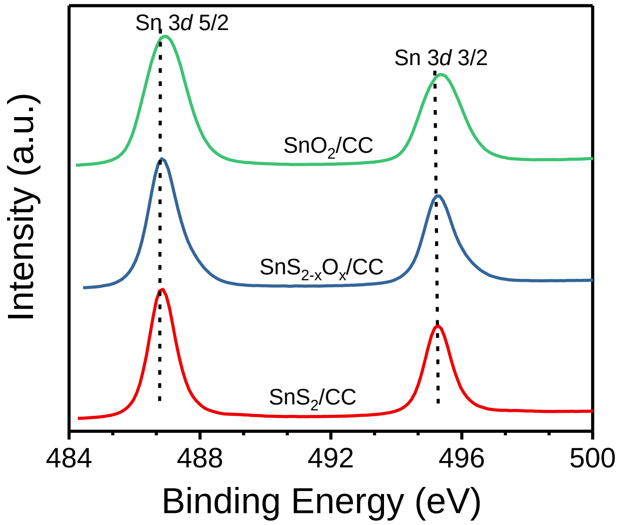

Fig. S8 Sn 3*d* XPS spectra of pristine SnS_2, $SnS_{2-x}O_x$, and SnO_2

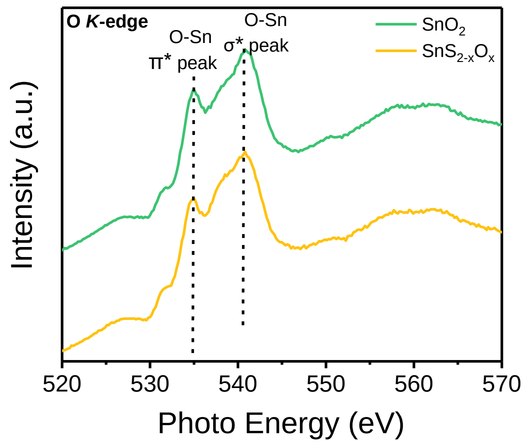

Fig. S9 O *K*-edge XAS spectra of $SnS_{2-x}O_x$ and SnO_2

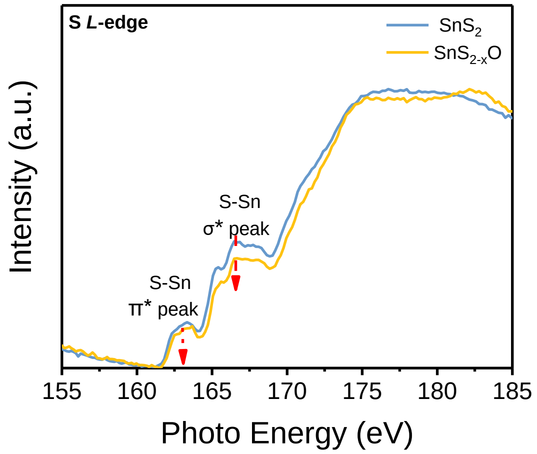

Fig. S10 S *L*-edge XAS spectra of SnS_2 and $SnS_{2-x}O_x$

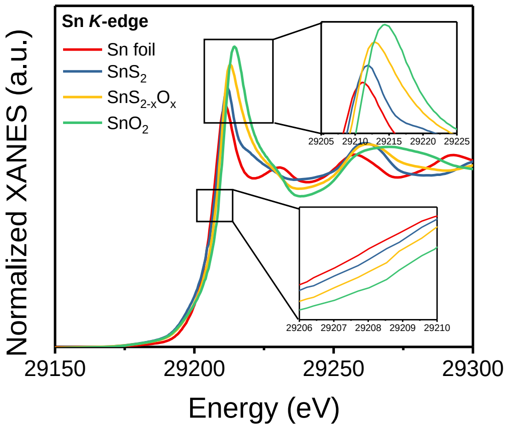

Fig. S11 Normalized XANES spectra of Sn *K*-edge for Sn foil, SnS_2, $SnS_{2-x}O_x$, and SnO_2

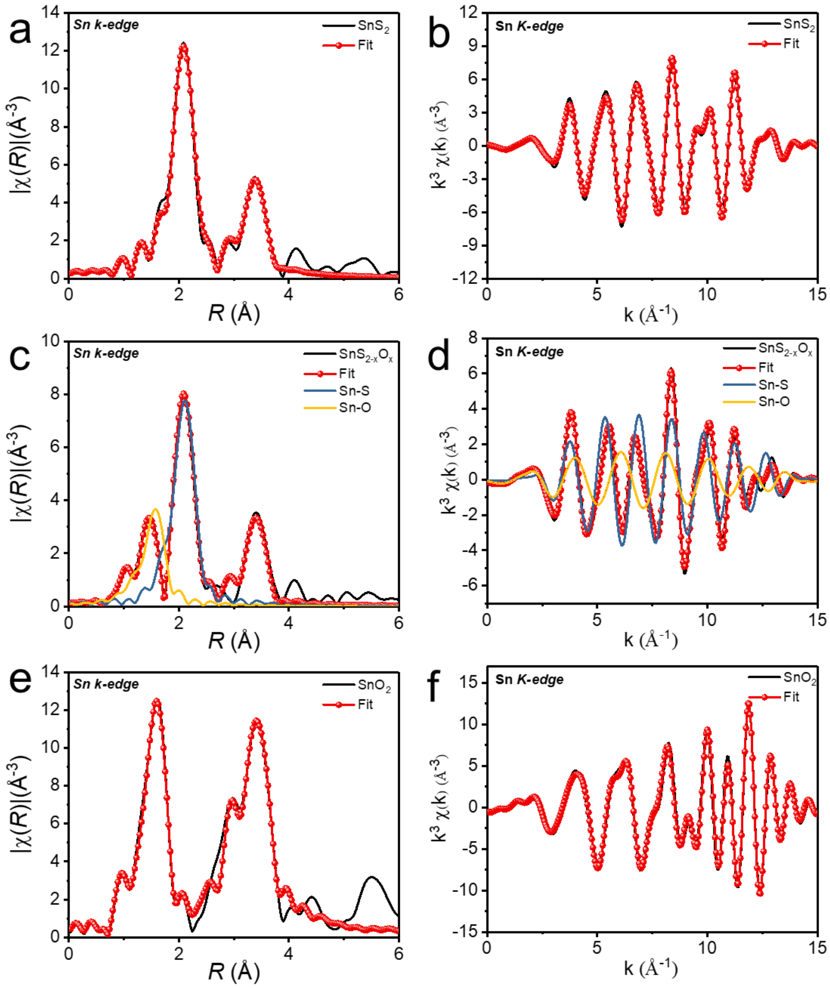

Fig. S12 Synchrotron radiation XAFS measurements. The Fourier transforms FT($k^3X(k)$) of the extended X-ray absorption fine structure (EXAFS) for Sn K-edge of the pristine SnS_2 (**a**), $SnS_{2-x}O_x$ (**c**), and SnO_2 (**e**). Sn K-edge EXAFS oscillation function $k^3X(k)$ for the pristine SnS_2 (**b**), $SnS_{2-x}O_x$ (**d**), and SnO_2 (**e**)

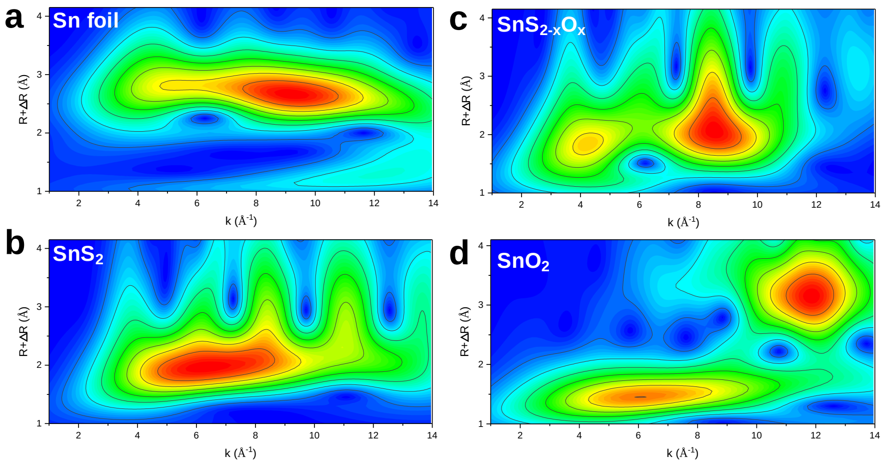

Fig. S13 Wavelet transform (WT) of Sn foil, SnS_2, $SnS_{2-x}O_x$, and SnO_2, respectively

Table S1 Best fitting EXAFS data for pristine SnS_2, $SnS_{2-x}O_x$, and SnO_2

Sample	Path	CN	R(Å)	σ^2 (10^{-3} Å^2)	ΔE_0 (eV)
SnS_2	Sn-S	6.0	2.55	3.8	2.5
$SnO_{2-x}O_x$	Sn-S	4.3	2.56	5.0	4.7
	Sn-O	2.1	2.04	3.6	0.3
SnO_2	Sn-O	6.0	2.09	4.1	-2.7

Note: For SnS_2: K-range: 2.22-13.20; R-range: 1.47-3.87; amp: 0.85; For $SnO_{2-x}O_x$: K-range: 2.36-13.61; R-range: 1.20-3.87; amp: 0.85; For Ag: K-range: 2.31-14.01; R-range: 1.09-3.90; amp: 0.90; *CN*; coordination number; R, bonding distance; σ^2, Debye-Waller factor; ΔE_0, inner potential shift.

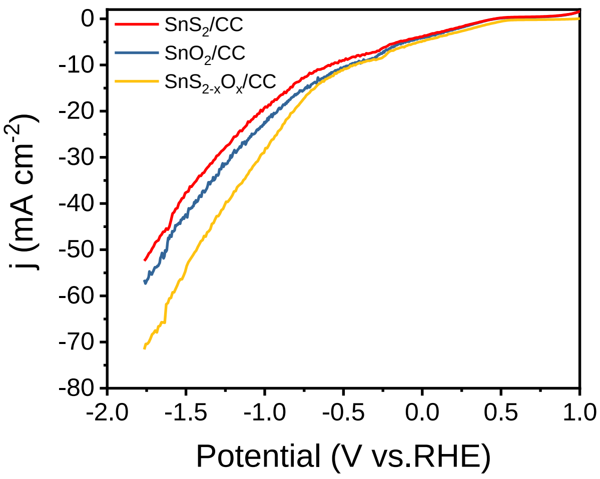

Fig. S14 Linear sweep voltammetry curves (LSV) in the CO_2 saturated 0.5 M $KHCO_3$ aqueous solution for pristine SnS_2/CC, $SnS_{2-x}O_x$/CC, and SnO_2/CC

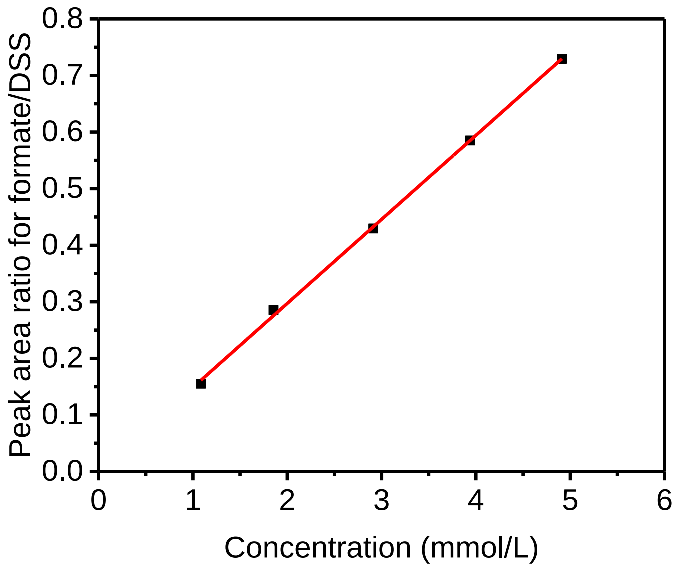

Fig. S15 Plot of concentration with peak-area ratio of HCOOH/DSS. The standard curve showed good linear relation of peak-area ratio for HCOOH/DSS with HCOOH concentration (y= 0.1485x, R^2= 0.9996).

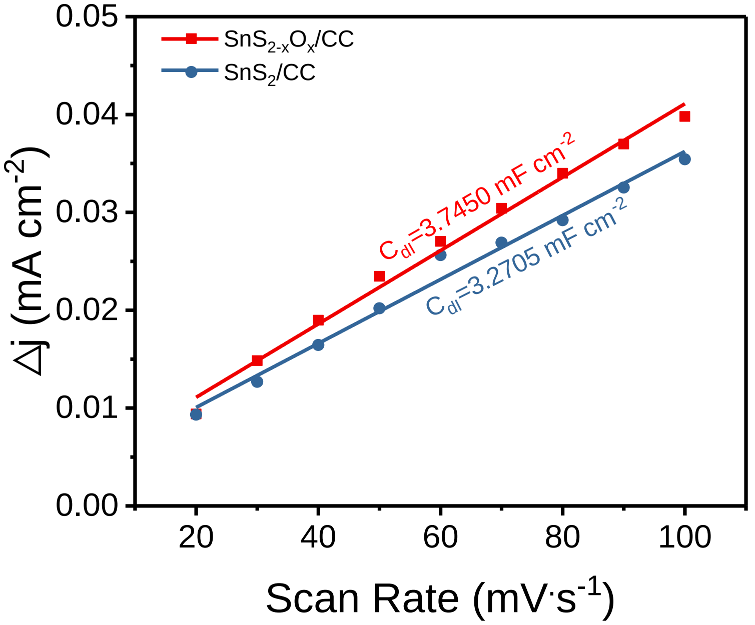

Fig. S16 Charging current density differences plotted against scan rates for pristine SnS_2/CC, $SnS_{2-x}O_x$/CC

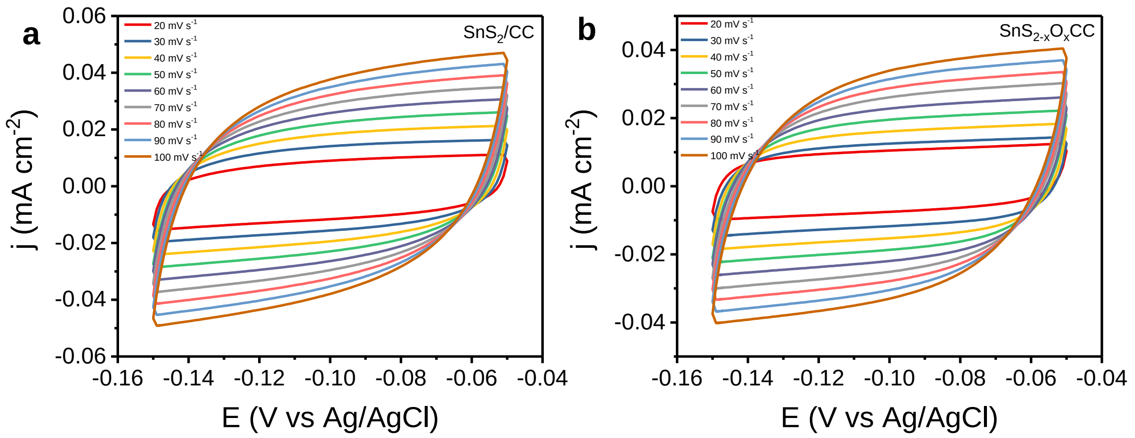

Fig. S17 CV curves of (a) pristine SnS_2/CC and $SnS_{2-x}O_x$/CC with various scan rates

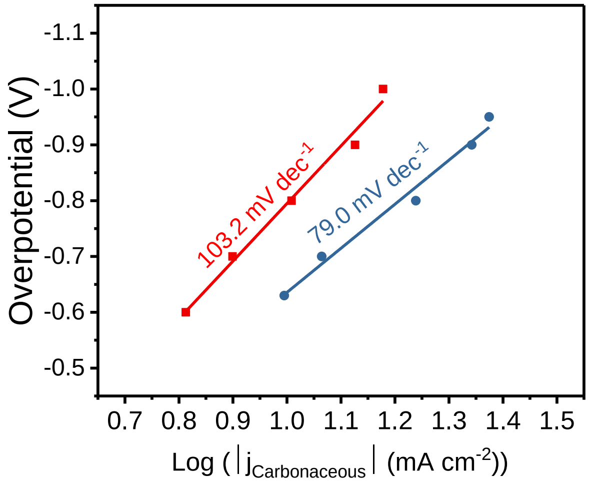

Fig. S18 Tafel plots of pristine SnS_2/CC and $SnS_{2-x}O_x$/CC

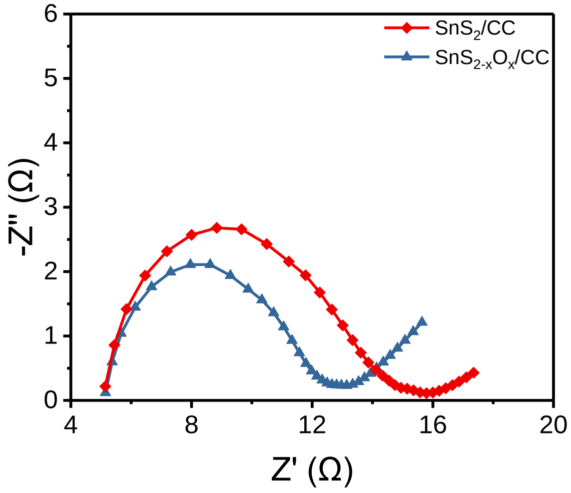

Fig. S19 Nyquist plots of pristine SnS_2/CC and $SnS_{2-x}O_x$/CC

Table S2 Structural parameters of $SnS_{2-x}O_x$/CC at the Sn *K*-edge extracted from quantitative EXAFS curve-fittings using the ARTEMIS module of IFEFFIT

Sample	Path	CN	R(Å)	σ^2 (10^{-3} Å^2)	ΔE_0 (eV)
OCV	Sn-S	3.9	2.57	4.3	4.9
	Sn-O	2.5	2.03	4.5	-2.9
-0.4	Sn-S	2.5	2.57	5.3	8.4
	Sn-O	3.6	2.05	3.07	2.5
-0.9	Sn-S	2.5	2.58	7.8	9.3
	Sn-O	4.2	2.05	4.1	5.3
After reaction	Sn-S	2.5	2.58	7.5	9.6
	Sn-O	3.6	2.05	3.0	2.7

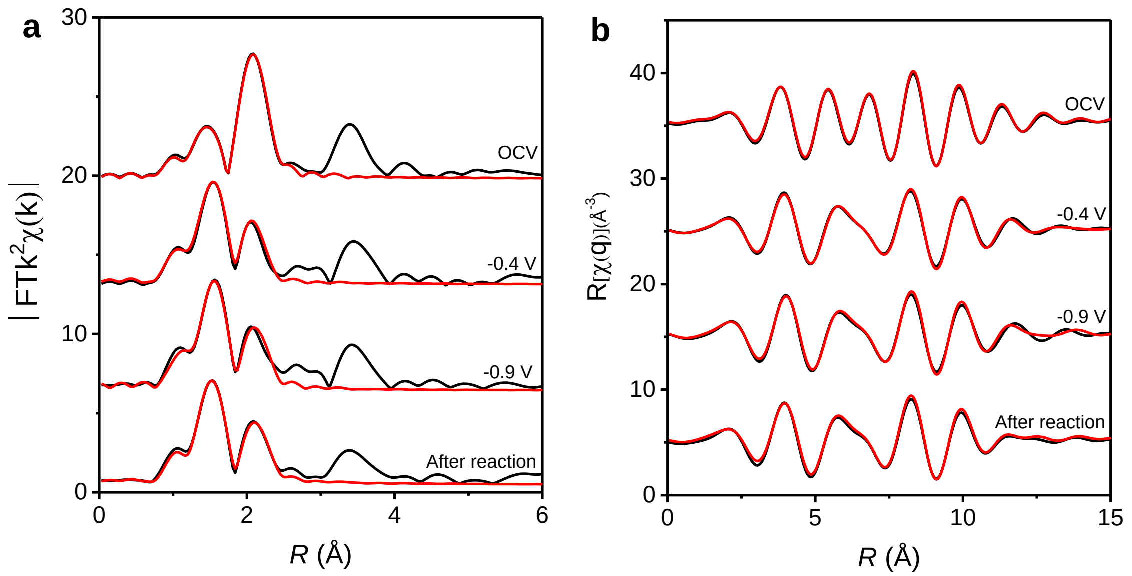

Fig. S20 (**a**) Least-squares curve-fitting analysis of operando EXAFS spectra at the Sn K-edge (**b**) Corresponding Re($k^2\chi$(k)) oscillations

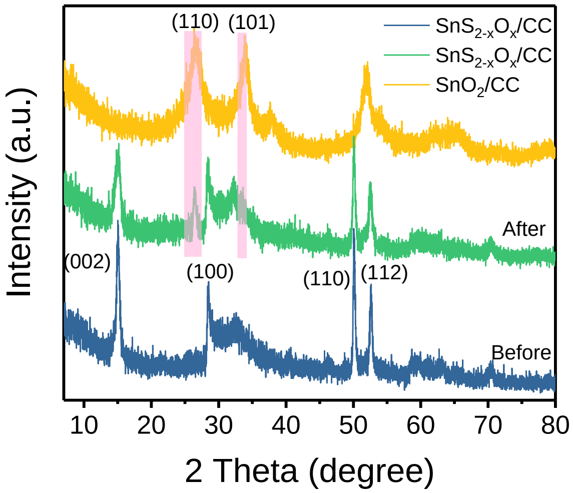

Fig. S21 XRD patterns of $SnS_{2-x}O_x$/CC before and after reaction

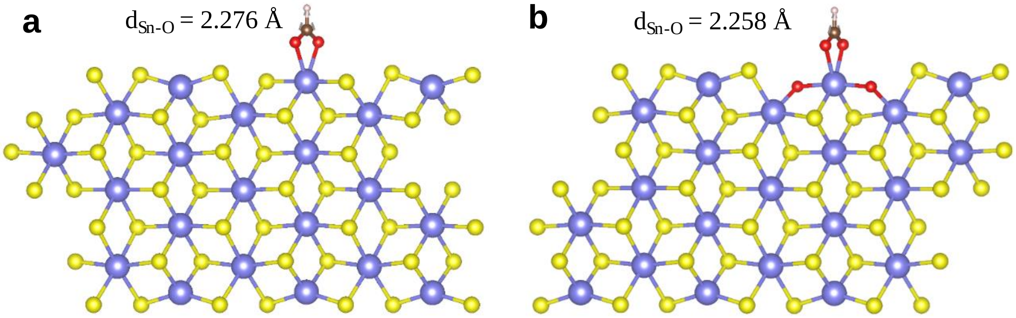

Fig. S22 Optimized adsorption configurations of HCOO* intermediates on the surface of the pristine SnS_2 slab (**a**) and $SnS_{2-x}O_x$ slab (**b**)

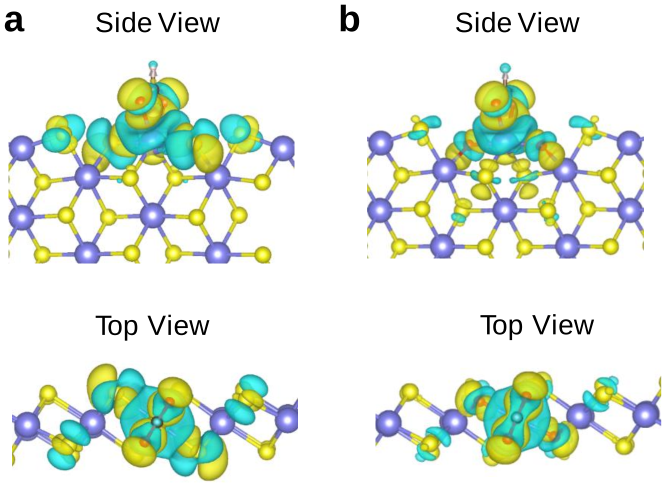

Fig. S23 Electron density difference plot of the HCOO* intermediate adsorption structure for pristine SnS_2 slab (**a**) and $SnS_{2-x}O_x$ slab (**b**). Yellow contours indicate electron accumulation and light green contours denote electron deletion.
